# Associations between prolonged second stage of labor and maternal and neonatal outcomes in freestanding birth centers: a retrospective analysis

**DOI:** 10.1186/s12884-022-04421-8

**Published:** 2022-02-04

**Authors:** Nancy A. Niemczyk, Dianxu Ren, Susan R. Stapleton

**Affiliations:** 1 Department of Health Promotion and Development, School of Nursing, University of Pittsburgh, 3500 Victoria Street, 440 Victoria Building, Pittsburgh, PA 15261 USA; 2 Center for Research and Evaluation, School of Nursing, University of Pittsburgh, 3500 Victoria Street, 440 Victoria Building, Pittsburgh, PA 15261 USA; 3 American Association of Birth Centers, 3123 Gottschall Road, Perkiomenville, PA 18074 USA

**Keywords:** Labor, Labor and delivery, Maternal and fetal outcome, Neonatal intensive care unit, Prolonged second stage, Second stage, Third- and fourth-degree laceration

## Abstract

**Background:**

Current guidelines for second stage management do not provide guidance for community birth providers about when best to transfer women to hospital care for prolonged second stage. Our goal was to increase the evidence base for these providers by: 1) describing the lengths of second stage labor in freestanding birth centers, and 2) determining whether proportions of postpartum women and newborns experiencing complications change as length of second stage labor increases.

**Methods:**

This study is a retrospective analysis of de-identified client-level data collected in the American Association of Birth Centers Perinatal Data Registry, including women giving birth in freestanding birth centers January 1, 2007 to December 31, 2016. We plotted proportions of postpartum women and newborns transferred to hospital care against length of the second stage of labor, and assessed significance of these with the Cochran-Armitage test for trend or chi-square test. Secondary maternal and newborn outcomes were compared for dyads with normal and prolonged second stages of labor using Fisher’s exact test.

**Results:**

Second stage labor exceeded 3 hours for 2.3% of primiparous women and 2 hours for 6.6% of multiparous women.

Newborn transfers increased as second stage increased from < 15 minutes to > 2 hours (0.6% to 6.33%, p for trend = 0.0008, for primiparous women, and 1.4% to 10.6%, p for trend < 0.0001, for multiparous women.) Postpartum transfers for multiparous women increased from 1.4% after second stage < 15 minutes to greater than 4% for women after second stage exceeding 2 hours (p for trend < 0.0001.)

**Conclusions:**

Complications requiring hospitalization of postpartum women and newborns become more common as the length of the second stage increases. Birth center guidelines should consider not just presence of progress but also absolute length of time as indications for transfer.

## Background

While the majority of women who experience long second stages of labor will give birth vaginally without major complications for themselves or their newborns, both maternal and neonatal complications are more frequent after a prolonged second stage [1–3]. Complications for women include intensive care unit admission, postpartum hemorrhage, episiotomy, 3^rd^ or 4^th^ degree perineal laceration, chorioamnionitis, and endometritis [3–6]. Newborn risks include asphyxia, neonatal intensive care unit admissions, sepsis, seizures, and low Apgar scores [1, 3, 4, 7, 8]. In 2014, the American College of Obstetricians and Gynecologists and the Society for Maternal-Fetal Medicine issued a consensus statement on safe prevention of the primary cesarean delivery [9]. This statement recommends that absent maternal or fetal indications for cesarean, women may push for at least 3 hours for a first birth and 2 hours for a subsequent birth before providers perform an operative delivery for arrest of descent. Longer second stages might be appropriate based on individual circumstances, including the use of neuraxial analgesia. This new guideline increased by an hour the time many women were given for the second stage of labor, and widely affected clinical practice [10].

The consensus statement, however, did not address all the needs of providers who attend births in community settings. Midwives attending community births (births occurring at home or in a freestanding birth center) must decide not only when a woman might need a cesarean for arrest of the second stage, but also whether there is a time when she is safer laboring in the hospital, even though she may continue to push. The consensus guidelines offer no guidance for this. Additionally, the guidelines are based solely on research done in hospital settings, limiting their generalizability to births at home or in freestanding birth centers. Freestanding birth centers are facilities separate from hospitals designed to facilitate physiologic (spontaneous, no augmentation or pharmacologic analgesia) birth [11, 12]. The safety of birth in accredited freestanding birth centers has been well-established [13–16] and the percentage of births in the United States occurring outside the hospital increased 85%, to 1.61% of births, between 2004 and 2017 [17]. The Commission for the Accreditation of Birth Centers (CABC), the national accrediting body for freestanding birth centers, in the absence of evidence, has been unable to set specific guidelines on appropriate length of second stage labor in birth centers. It requires only that birth centers should “include guidelines for management of prolonged first and second stage labor that are consistent with best-available evidence.” Previous research from our group found that midwives practicing in freestanding birth centers use a multifactorial, multisensory assessment of progress in the second stage of labor, with time passed only one factor considered [18].

Consequently, birth center staff are in the position of devising evidence-based practice guidelines for prolonged second stage labor without national guidelines addressing their practice site or research done in their setting. We designed our project to expand the evidence base about safety of prolonged second stage labor in community settings by describing the frequency and outcomes of prolonged second stages as currently managed in US birth centers. Our specific objectives were to: 1) determine the lengths of the second stage of labor for births occurring in freestanding birth centers, and 2) describe whether the proportions of postpartum women and newborns who experience complications, including transfer to hospital care, change as length of second stage labor increases.

## Materials and methods

### Study Design & Population

This study is a retrospective analysis of de-identified client-level data collected in the American Association of Birth Centers (AABC) Perinatal Data Registry v.2.0 and 3.0 ^TM^ (PDR). Currently, 102 birth centers voluntarily contribute data to the PDR, representing approximately 1/3 of known US birth centers. Contributing birth centers enter data on all pregnant clients, beginning with the first pregnancy visit, and continue data collection through postpartum care for all clients who remain in care. Staff enter data into the PDR prospectively, and quality assurance mechanisms ensure systematic client enrollment, timely completion of data, minimization of loss to follow-up, and data consistency [19, 20]. Data included consist of demographic, descriptive, and process and outcome indicators. Registry protocols adhere to guidelines from the Agency for Healthcare Research and Quality [21]. A 2010 validation study found 97.1% concordance between PDR and health records for 29 key variables [20].

In the United States, freestanding birth centers are primarily regulated at the state level. Some states prescribe clear guidelines for choice of appropriate clients and specific criteria for hospital transfer, some have more vague criteria, and some do not regulate freestanding birth centers. Accreditation by CABC requires adherence to some common guidelines but is optional in most states. Clinical practice guidelines differ among birth centers, but common elements include care largely provided by licensed midwives, admission of women at term free of significant complications, exclusive use of intermittent auscultation for fetal surveillance in labor, and support for physiologic birth. Accreditation does not require specific management guidelines for the third stage of labor, other than prohibiting manual placenta removal unless the client is unstable for transport after use of first-line interventions for hemorrhage, but CABC standards do state that active management of the third stage with prophylactic administration of intramuscular oxytocin is appropriate in birth centers [22]. Birth center midwives work within a system allowing for collaborative care or referral for women requiring a higher level of care than the birth center provides. To assess comparability of care in different birth centers we collected practice guidelines for the second stage labor from centers contributing study data.

The sample included women who gave birth in a freestanding birth center contributing data to the PDR between January 1, 2007 and December 31, 2016. The inclusion criterion was documented length of the second stage. Exclusion criteria included multiple gestation, non-vertex presentation, prior cesarean birth, newborns with major congenital anomalies, and known fetal demise prior to birth center admission. Women signed a written consent form permitting inclusion of their data in the registry and its use for research purposes. As this was an analysis of preexisting de-identified data, the Institutional Review Board at the University of Pittsburgh, Human Research Protection Office, deemed the study exempt from approval. All study methods were in accordance with the relevant institutional and national ethics guidelines.

### Variables

The primary exposure was length of second stage labor, categorized in the PDR as less than 15 minutes, 15-30 minutes, 31-60 minutes, 61-90 minutes, 91 minutes to <2 hours, 2-3 hours, 4-5 hour and greater than 5 hours. We also categorized second stage labor as normal or prolonged according to the consensus guidelines, as greater than 3 hours for nulliparous women and greater than 2 hours for parous women. When comparing indications for transfer, there were too few transfer in the prolonged second stage group to perform significance testing, so we compared transfers for second stages lasting more and less than 2 hours for first births and for second stages lasting more and less than 1 hour for subsequent births. Instructions for the PDR define length of second stage as “Time from full cervical dilation to birth of infant,” with further instructions for people entering data that, “If you do not know the exact time patient was fully dilated, use the time she began to push spontaneously or the time she was found to be fully dilated.” Our previous qualitative research on management of second stage in birth centers found that many birth center midwives rarely performed cervical exams to confirm cervical dilatation before pushing, and that onset of second stage is often a retrospective diagnosis often based on signs including spontaneous pushing [18].

The primary outcomes were maternal postpartum and newborn transfers. Postpartum transfers were defined as transfer of the postpartum woman to a hospital after the birth for a medical reason, and newborn transfers as transfer of the newborn to a hospital for a medical reason. These serve as natural composite outcomes, because women and newborns are transferred to the hospital for management of any severe complication. Situations in which a postpartum woman or newborn was admitted to the hospital for a non-medical reason (such as a power outage in the birth center, or to accompany a mother or newborn transferred for a medical reason) were not considered transfers.

As secondary outcomes for women, we considered hemorrhage (estimated blood loss greater than 500 cc), active management of the third stage of labor (coded as yes/no), retained placenta (requiring manual removal), fever (greater than 38^0^C), 3^rd^ or 4^th^ degree perineal lacerations, and indication for postpartum transfer. Indications for postpartum transfer included hemorrhage and retained placenta as above, hematoma, and repair of episiotomy or laceration, which included any laceration or episiotomy of any degree unable to be repaired by the provider or in the birth center setting. Secondary outcomes for newborns included use of positive pressure ventilation, endotracheal intubation, antibiotic administration, diagnosis of sepsis, intrapartum and neonatal deaths, and indication for newborn transfer.

### Statistical Analysis

All statistical analyses were stratified by parity (first vs. subsequent births.) Summary statistics were presented as means and standard deviations or numbers and percentages, as appropriate. For primary outcomes, we plotted proportions of postpartum women and newborns transferred to hospital care against length of second stage of labor, and assessed significance either with the Cochran-Armitage test for trend if the relationship appeared linear or with the chi-squared test if it did not. Proportions of secondary outcomes were compared for dyads with normal and prolonged second stages of labor using Fisher’s exact test. Indications for transfers for dyads with shorter and longer second stage of labor were compared using the chi-squared test. Given the exploratory nature of our secondary outcomes, we did not adjust for multiple comparisons. Analysis was performed using SAS statistical software release 9.4 (SAS Institute, Cary, NC). Clinical practice guidelines for different birth centers were categorized by content.

## Results

**Fig. 1:**
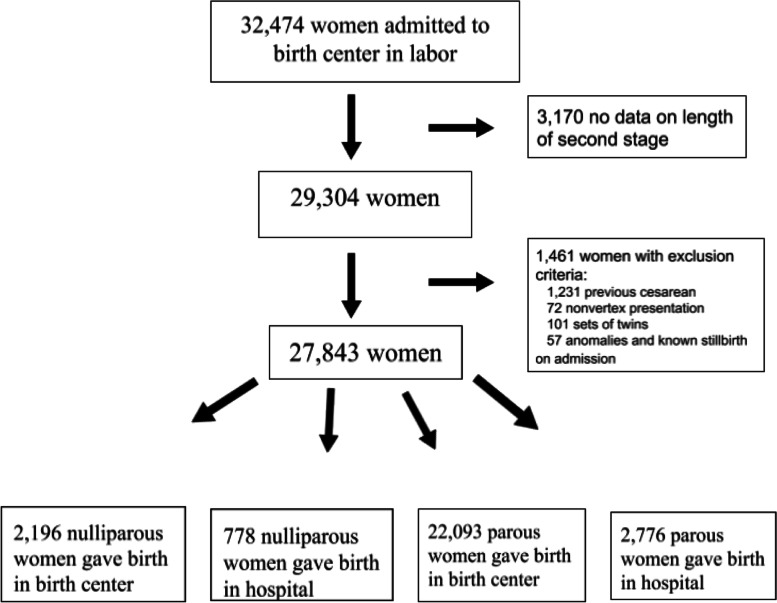
Participant flow

The original dataset consisted of 34,097 births. After eliminating labors with exclusion criteria and missing data about the length of the second stage, 27,843 births remained. The analytic sample consisted of 2196 first births and 22,093 births to parous women occurring in birth centers (Fig. 1). The remaining 3554 women gave birth in the hospital and were not included in the analysis, but will be included in a future manuscript exploring whether earlier hospital transfer results in better maternal or fetal outcomes.

Demographic information is found in Table 1. Participants in the analysis were primarily white (78.4% nulliparous, 83.2% parous) and had a mean age of 28.6 ± 5.1 years for nulliparous women and 29.5 ± 4.8 years for parous women. Prolonged second stage (> 3 hrs in nulliparous and >2 hrs in parous women) was experienced by 2.3% of nulliparous women and 6.6% of parous women.

**Table 1:** Characteristics of women giving birth in birth center

	Nulliparous (*n*=2196)	Parous (*n*=22,093)
Age	28.6 (5.1)	29.5(4.8)
BMI	23.9 (4.1)	24.2 (4.7)
Race		
White	1720 (78.4 )	18,385 (83.2)
Black	150 (6.8)	1139 (5.2)
Asian	47 (2.1)	375 (1.7)
Other and multiracial	208 (9.5)	1603 (7.3)
Missing race	69 (3.1)	534 (2.4)
Hispanic ethnicity	184 (8.4)	1802 (8.2)
Public insurance	683 (31.1)	5790 (26.2)
Second stage > 1 hour	742 (43.9)	2858 (17.6)
Second stage > 2 hours	276 (16.3)	1071 (6.6)
Second stage > 3.5 hours	39 (2.3)	194 (1.2)

Mean (SD) or n (%)

**Fig. 2:**
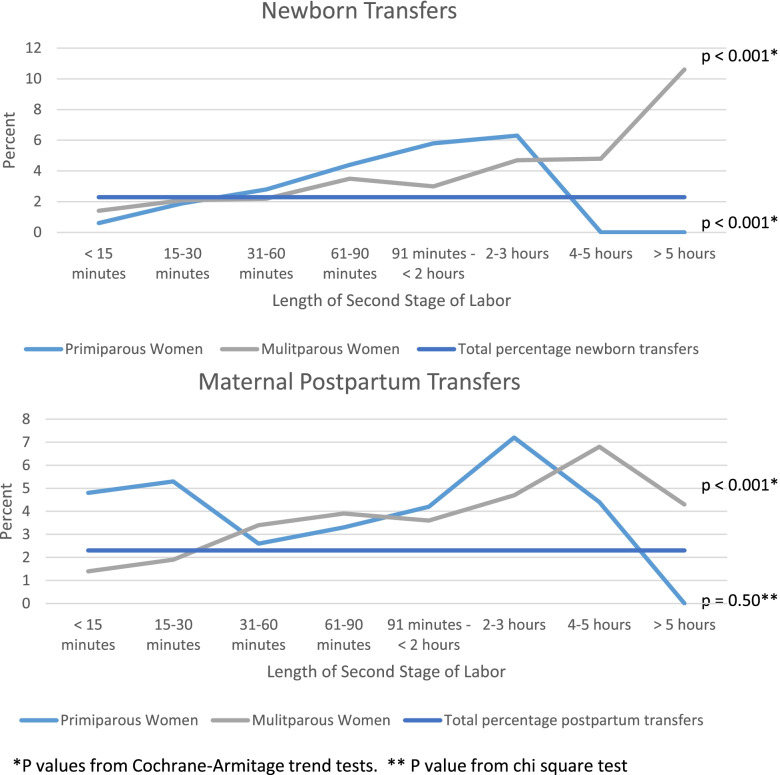
Newborn and maternal postpartum transfers

Proportions of postpartum women and newborns transferred to the hospital for complications are presented in Fig. 2. Overall, 2.3% of postpartum women and 2.3% of newborns transferred to the hospital for medical indications (represented by the horizontal line.) Transfers for all newborns and for postpartum multiparous women increased linearly as the length of second stage increased. For newborns of primiparous women, transfers increased from 0.6% of newborns born after second stage labors less than 15 minutes to 6.3% for newborns born after second stage labors of 2 to 3.5 hours (p for trend = 0.0008.) For newborns of multiparous women, transfers increased from 1.4% for newborns born after second stage labors shorter than 15 minutes to 10.6% for newborns born after second stages longer than 5 hours (p for trend < 0.0001.) Postpartum transfers for multiparous women increased from 1.4% for women whose second stage of labor was less than 15 minutes to greater than 4% for women with second stage of labor exceeding 2 hours (p for trend < 0.0001.) There were no significant differences in postpartum transfers for primiparous women, although it appears that there is a j-shaped relationship, with the fewest women requiring transfer after second stage labors of 31 to 90 minutes.

Complications were more common after prolonged second stages than after normal second stages for women giving birth in birth centers (Table 2.) These differences were not statistically significant for primiparous women and their newborns, but were for multiparous women and their newborns. Multiparous women with second stage labors over 2 hours were more likely to be experience postpartum hemorrhage (15.0% vs 9.7%, P<0.001), retained placenta (2.5% vs 1.2%, *p*=0.005), maternal fever (0.7% vs. 0.3%, *p*=0.03), and severe perineal lacerations (3.9% vs. 0.9%, p<0.001.) Similarly, newborns born to multiparous women after a second stage greater than 2 hours were more likely to require positive pressure ventilation (0.3% vs 0.04%, *p*=0.02), and be diagnosed with sepsis (1.2% vs 0.4%, *p*=0.002.) Perinatal deaths were few, but were more common for newborns of multiparous women born after second stages of labor exceeding 2 hours (1 intrapartum death (0.1%) vs. 0 (0.0%), 2 neonatal deaths (0.2%) vs. 3 (0.02%), *p*=0.005.)

**Table 2:** Maternal and newborn outcomes after prolonged second stage of labor

	Primiparous Women			Multiparous Women		
Length of Second Stage	≤ 3 hours	> 3hours	*P* value	≤ 2 hours	> 2 hours	*P* value
**Maternal Outcomes:**						
Hemorrhage	157/1391 11.3%	5/31 16.1%	0.39	1185/12,284 9.7%	127/846 15.0%	<0.001
Active 3^rd^ Stage Management	463/1630 28.4%	12/39 30.8%	0.72	4332/14,628 29.6%	371/1003 37.0%	<0.001
Retained placenta	33/1363 2.4%	1/31 3.2%	0.54	145/12,094 1.2%	20/811 2.5%	0.005
Maternal fever	7/1355 0.5%	0/31 0.0%	>0.99	32/12,067 0.3%	6/809 0.7%	0.03
3^rd^ or 4^th^ degree laceration	27/1358 1.6%	0/31 0.0%	>0.99	132/12,095 0.9%	42/814 3.9%	<0.001
**Newborn Outcomes:**						
Use of positive pressure ventilation	3/1652 0.2%	0/39 0.0%	>0.99	6/15,173 0.04%	3/1071 0.3%	0.02
Newborn intubation	0/1652 0.0%	0/39 0.0%	-	2/15,173 0.01%	0/1071 0.00%	>0.99
Newborn antibiotics	21/1652 1.3%	0/39 0.0%	>0.99	47/15,173 0.3%	10/1071 0.9%	0.004
Newborn sepsis	21/1652 1.3%	0/39 0.0%	>0.99	65/15,173 0.4%	13/1071 1.2%	0.002
Perinatal deaths			>0.99			0.005
Intrapartum death	0/1652 0.0%	0/39 0.0%		0/15,170 0.0%	1/1071 0.1%	
Neonatal death	1/1652 0.1%	0/39 0.0%		3/15,170 0.02%	2/1071 0.2%	

Significance testing done with Fisher’s Exact Test. Denominators differ due to presence of missing data

Indications for transfers were different for multiparous women (p<0.01) and their newborns (p<0.01) after longer second stages (Table 3). Repair of laceration or episiotomy was the indication for 23.6% of postpartum maternal transfers for multiparous women after a second stage under 1 hour in length, but for 44.5% of those whose second stages exceeded 1 hour. A greater proportion of transfers were for respiratory issues in newborns of multiparous women after second stages exceeding 1 hours than for shorter second stages (74.1% vs. 62.5%.)

**Table 3:** Indications for maternal postpartum and newborn transfers

	**First Births**		Subsequent Births^*^	
**Indication for postpartum transfer**	**Second stage < 2 hours*n*=55**	**Second stage > 2 hours*n*=18**	**Second stage < 1 hour** ***n*=259**	**Second stage > 1 hour*n*=121**
Hemorrhage	14 (25.5%)	7 (38.9%)	79 (30.5%)	25 (20.7%)
Retained placenta	15 (27.3%)	3 (16.7%)	68 (26.3%)	24 (19.8%)
Laceration or episiotomy repair	17 (30.9%)	6 (33.3%)	61 (23.6%)	55 (44.5%)
Hematoma	6 (10.9%)	1 (5.6%)	8 (3.1%)	1 (0.8%)
Other	3 (5.5%)	1 (5.6%)	43 (16.6%)	16 (13.2%)
	**First Births**		Subsequent Births^*^	
**Indication for newborn transfer**	**Second stage < 2 hours** **n=43**	**Second stage > 2 hours** **n=15**	**Second stage < 1 hour n=232**	**Second stage > 1 hour** **n=112**
5 minute apgar score < 7	6 (14.0%)	1 (6.7%)	19 (8.2%)	14 (12.5%)
Respiratory problems	30 (69.8%)	14 (93.3%)	145 (62.5%)	83 (74.1%)
Anomaly	2 (4.7%)	0 (0.0%)	5 (2.2%)	0 (0.0%)
Other	5 (11.6%)	0 (0.0%)	63 (27.2%)	15 (13.4%)

Data presented as N (%)

Percentage is percentage of total transfers

*p < 0.01 per chi square test

Thirty birth centers provided clinical practice guidelines for management of second stage labor, and these varied considerably. Eight birth centers had no written guidelines for when to transfer women to the hospital for prolonged second stage. Guidelines for nulliparous women ranged from transfer after 1 hour of second stage with no progress (2 birth centers) to transfer after 4 hours without progress (3 birth centers.) Guidelines for parous women ranged from transfer after 1 hour without descent (3 birth centers) to transfer after 3 hours if birth not imminent (6 birth centers.) Most guidelines included an element of subjectivity, enabling providers to determine what qualified as “change” or “progress.”

## Discussion

The goals of our research were to describe the frequency and outcomes of prolonged second stage of labor in freestanding birth centers, under current practice conditions. We found that 2.3% of healthy nulliparous women giving birth in freestanding birth centers, and 6.6% of parous women, had prolonged second stages of labor. Even in this low-risk group of women, as the length of the second stage of labor increased, transfers for complications were significantly more frequent for all newborns, and maternal postpartum transfers for complications were significantly more frequent for multiparous women. For multiparous women, hemorrhage, retained placenta, maternal fever, and severe perineal lacerations were more common with longer second stages of labor; for their newborns, use of positive pressure ventilation and sepsis were more common. Hemorrhage and retained placenta were more common for multiparous women despite more frequent use of active third stage management for women with prolonged second stage of labor, suggesting use of active third stage management was suboptimal in this sample. Indications for transfers also differed with longer second stages for multiparous women; higher proportions of maternal postpartum transfers were for repair of severe laceration and higher proportions of newborn transfers were for respiratory problems.

Incidence of some of the outcomes in the study differ from generally quoted incidence of these complications, likely reflecting distinctive aspects of the population. Reported postpartum hemorrhages in our dataset, between 9.7% and 16.1% depending on group, are higher than nationally reported incidence of 1 to 5% [23]. In this dataset, postpartum hemorrhage was defined as estimated blood loss of greater than 500 cc, as opposed to the new, more stringent definition of blood loss greater than 1000 cc. Additionally, birth centers primarily reported estimated blood losses, which are less accurate than quantitative blood losses [24]. As average blood loss from vaginal birth is approximately 500 cc, these percentages of women exceeding 500 cc seem realistic [25]. Fewer than 10% of women reported with blood losses greater than 500 cc required transfer to the hospital for care, supporting that the majority of cases of hemorrhage had estimated blood loss slightly greater than 500cc and did not result in substantial morbidity. Studies of home birth have found similar incidence of blood loss greater than 500 cc [26]. The incidence of maternal postpartum fever of 0.0 to 0.5% is lower than the generally quoted 1-4% incidence of chorioamnionitis, but reflects that birth center midwives will transfer women to the hospital who exhibit fever during labor [27]. Few newborns required positive pressure ventilation, but we were unable to determine frequency of positive pressure ventilation in a similar low risk group.

This is the first study demonstrating that for births in a community setting, postpartum and neonatal complications requiring transfer to the hospital are more common as the length of the second stage of labor increases. Consistent with data from hospital studies, even in this low risk, low-intervention population, maternal [1, 8, 28–31] and newborn [1, 8, 28, 29] complications were higher with longer second stages. Apparent lower rates of transfer among primiparous women with second stages longer than 4 hours are likely due to small numbers of women in this group. There were significantly more perinatal deaths (intrapartum and neonatal) for multiparous women with second stage labors longer than 2 hours, but given small numbers, (3 deaths each for second stages shorter and longer than 2 hours), this should be interpreted cautiously. These outcomes occurred despite the fact that midwives transferred women to the hospital during labor if they developed risk factors that made the midwives think that the hospital was the more appropriate birth site.

Our finding that indications for transfer change for multiparous women and their newborns after longer second stage labor is novel, but consistent with research performed in hospital settings. A higher proportion of women transferred for repair of severe perineal lacerations after a long second stage; this is consistent with hospital studies finding that these lacerations are more common with prolonged second stage [1, 8, 29]. Similarly, more newborn transfers were for respiratory issues after prolonged second stage. This is consistent with hospital studies finding that newborns born after prolonged second stage have higher incidence of low Apgar scores, umbilical artery pH less than 7, need for resuscitation, and intensive care unit admission [1, 29].

This study should not be interpreted as making general statements about complications of prolonged second stage, which have been well-established. The sample we analyzed was highly censored. Women who began labor in the birth center transferred to the hospital during both the first and second stages for various indications, including prolonged second stage of labor itself. Thus, we cannot make general statements about outcomes of prolonged second stage. Rather, results should be interpreted in light of the aims of the study, to describe outcomes of prolonged second stage as it currently occurs in freestanding birth centers. When interpreted in this light, its value is highlighting that even when midwives follow clinical practice guidelines and use their best clinical judgment to transfer women at high risk, complications are still more frequent in women with prolonged second stages.

This study represents the first time birth center providers have data from their practice setting to use for development of clinical practice guidelines. We found that most birth centers have practice guidelines for management of the second stage of labor that include transfer to the hospital setting after a certain length of time *without progress*. Given the findings that even with continually progressive second stages resulting in spontaneous vaginal births, medical indications for postpartum and newborn transfer to a hospital increase with longer second stage of labor, birth center providers and their consulting physicians should consider guidelines that include transferring women to hospital after a fixed number of hours of pushing. Consistent with research on prolonged second stage in hospitals, there does not appear to be an inflection point where outcomes worsen dramatically, but rather a steady increase in complications [3]. Precise guidelines for transfer timing would depend on distance from the hospital and a birth center’s individual circumstances, but midwives should be concerned about long second stages and a time frame between 2 and 3 hours for transfer might balance likelihood of safe vaginal birth with maternal and newborn risks.

In future analyses using this dataset we will explore further questions. The first is whether outcomes would be better if women were transferred to the hospital sooner during prolonged second stage of labor. For newborns, the most common indication for transfer was respiratory issues. These could potentially be mitigated by operative birth to end the second stage of labor and so might support earlier transfer. The most common indication for postpartum transfer for multiparous women was for repair of severe perineal lacerations. It is possible that perineal outcome for a spontaneous vaginal birth would be similar regardless of birth site, but exploration of this complication could provide information to guide clinical decision-making. A second area of research is whether we can identify characteristics that are associated with better or worse outcomes after prolonged second stage of labor. Perhaps better risk stratification could guide decision-making about transfers.

This study benefits from the use of a large, validated, national dataset that allows us to explore relatively rare outcomes. It has the limitations of any observational study based on a clinical dataset. While transfers, the primary outcomes, are required fields in the PDR and are consistently documented and have been validated [20], there is a substantial amount of missing data for the exposure (length of second stage) and some secondary outcomes, and these findings should be interpreted cautiously. Length of second stage was collected as a categorical variable, and the ranges go up to 1.5 hours. Thus, we do not know, for instance, how risk changed between 2 and 3.5 hours of second stage of labor. The highest category is for greater than 5 hours, not allowing us to explore outcomes of very long second stages of labor separately. Additionally, length of second stage may not have been collected consistently across all birth centers, as not all midwives perform vaginal exams to confirm full dilatation. It is unlikely, however, that any research protocol could precisely capture the moment of full dilatation to document onset of second stage labor. We believe that the possibility that there is some under-reporting of the length of second stage does not diminish the main results of this research. Since only approximately one third of birth centers contribute data to the PDR, birth centers that contribute data may differ in a systematic way from birth centers that do not contribute data. They may have more staff, which could facilitate data collection, or may be more connected to AABC, which could result in more use of AABC’s professional development resources to maintain current, high quality care. This potential for differences between birth centers that collect data and those that do not limits the generalizability of the study results.

## Conclusions

The second stage of labor can be long in freestanding birth centers, and while most women had good outcomes, complications requiring hospitalization of the postpartum woman and her newborn become more common as the length of the second stage increases. Birth center staff and their consulting physicians can use these results when developing evidence-based practice guidelines for birth centers, and should consider not just presence of progress but also the absolute amount of time as indications for transfer.

## Data Availability

The data that support the findings of this study are available from the American Association of Birth Centers. Forms for requesting data are available at www.birthcenters.org in the Research & Data section.
